# Measuring Failures Proneness: Scale Development and Preliminary Validations

**DOI:** 10.3389/fpsyg.2021.757051

**Published:** 2021-12-13

**Authors:** Irene Diamant, Zohar Rusou

**Affiliations:** ^1^School of Behavioral Sciences, The Academic College of Tel Aviv-Yaffo, Tel Aviv-Yafo, Israel; ^2^Department of Education and Psychology, The Open University of Israel, Ra’anana, Israel

**Keywords:** accident proneness, human error, personality, questionnaire development, safety

## Abstract

Behavioral failures can serve as precursors for accidents. Yet, individual differences in the predisposition to behavioral failures have predominantly been investigated within relatively narrow parameters, with the focus limited to subsets of behaviors or specific domains. A broader perspective might prove useful in illuminating correlations between various forms of accidents. The current research was undertaken as one step toward developing the concept of behavioral failures proneness in its multidimensional aspect. We report the initial stage of the development and validation of the Failures Proneness questionnaire (FP): a brief, multifaceted, self-report scale of common behavioral failures in everyday settings. In a preliminary phase we conceived an extensive pool of prospective items. Study 1 identified and validated the factor-structure of FP and reduced the scale to a brief measure of 16 items. Study 2 corroborated the factor structure of the FP and evaluated its construct validity by assessing its relationship with the Five Factor Model (FFM) of personality traits. Study 3 tested the criterion-related validity of the FP by assessing its ability to predict deviant behaviors. These studies provide evidence of the FP’s performance in generating valuable information on a broad range of behavioral antecedents of accidents.

## Introduction

Accidents occur in a variety of environments. In the majority, human failure is a key factor (McKenna, 1983; de Winter, 2014; Beus et al., 2015; Senders and Moray, 2020). The identification of behavioral failures as precursors to accidents could therefore aid in devising interventions for safety improvement (Lawton and Parker, 1998). Yet, individual differences in behavioral failures have predominantly been investigated within relatively narrow parameters, with the focus limited to subsets of behaviors (e.g., cognitive failure; Broadbent et al., 1982) or specific domains (e.g., traffic). Although these narrow perspectives have yielded insight into the important relationship between human failure and specific forms of accidents, a broader perspective might prove useful in illuminating the overall picture. Specifically, positive correlations which have been discovered between various forms of accidents (Salminen, 2005) raise a concern that links between various antecedents might have been neglected. The current research addressed this gap in the literature through the development and use of a Failures Proneness questionnaire (FP), a valid and reliable measuring instrument to assess individual differences in the predisposition for such failures. Such measures could enable systematic analysis that is imperative for guiding accident prevention attempts. To our knowledge, no tool such as this exists.

### Accidents and Human Failure

Human failure leads to accidents, which result in fatalities, heavy economic loss, and emotional distress. According to the International Labor Organization, on average, over 315 million accidents occur annually worldwide in workplaces alone, out of which about 2.3 million are fatal. Human failure causes extensive damage within health care systems (Makary and Daniel, 2016), software and computers (Kane, 2018), financial institutions (Leaver et al., 2018), transportation systems (World Health Organization, 2015), sports (Vanpoulle et al., 2017) and so forth. Significant efforts aimed at preventing accidents have been invested in improving work environments and in the development of control techniques. However, although these advances have reduced the net number of accidents, human failure has remained pervasive and its relative contribution to accidents has increased (McKenna, 1983; Lawton and Parker, 1998; de Winter, 2014; Beus et al., 2015). Consequently, researchers stress that the human factor should be highlighted (Wiegmann and Shappell, 2001; Celik and Cebi, 2009; de Winter, 2014).

### Accident Proneness

The term “accident proneness” conveys the notion that certain individuals are susceptible to accidents more than others with similar characteristics like age and gender (Visser et al., 2007). Individual differences in accident proneness have been a topic of great interest in ergonomics research since the beginning of the twentieth century. Then, the influential work of Greenwood and Woods (1919) revealed that a small percentage of workers in a British factory were implicated in most of the accidents that occurred. In the latter part of the twentieth century, accident proneness became a subject of much controversy, mainly due to theoretical and methodological deficiencies of early studies. Subsequent advances in methodology and conceptual frameworks, however, led to a renewed interest in the topic (Visser et al., 2007).

Over the past decades, increasing support for the presence and validity of accident proneness was obtained from several independent lines of research. Accumulated evidence showed that the number of individuals involved in repeated accidents was higher than would have been expected by chance (Visser et al., 2007), and that the tendency of individuals to be implicated in accidents is stable over time (Gauchard et al., 2003; Wåhlberg and Dorn, 2009; Dorn and af Wåhlberg, 2020). Moreover, ample studies demonstrated a significant correlation between accident liability and various personality traits. The negative association of Conscientiousness and Agreeableness with unsafe behaviors and accidents is well established in the literature on FFM personality traits (Arthur and Graziano, 1996; Cellar et al., 2001; Wallace and Vodanovich, 2003; Bogg and Roberts, 2004; Clarke and Robertson, 2005; Christian et al., 2009; Herzberg, 2009; Beus et al., 2015). Additional personal characteristics that have been linked to behavioral failures and accidents include impulsiveness, sensation and risk seeking (Jonah, 1997; Junge, 2000; Zuckerman, 2015; Zhang et al., 2019), emotional stability, a tendency toward anger (Sommer et al., 2008), cognitive deficits (Kessler et al., 2009; Vaa, 2014) and locus of control (internal vs. external – see Christian et al., 2009 for a review). In view of this vast body of evidence, an instrument for measuring individual differences in accident proneness might be useful for practitioners, by providing parameters according to which research outcomes could be compared and integrated (Iversen and Rundmo, 2002).

### Measuring Individual Differences in the Predisposition to Behavioral Failures

It would be reasonable to assume that individuals with a higher propensity for behavioral failures are at higher risk for accidents. A prominent construct that has been linked to accident proneness is “cognitive failure,” which denotes an overall propensity of an individual to experience slips and lapses in cognitive functioning and control (Broadbent et al., 1982). Numerous studies using the Cognitive Failures Questionnaire (CFQ) have suggested that cognitive failure is a largely stable trait-like construct that predicts involvement in accidents (Broadbent et al., 1982; Larson et al., 1997; Finomore et al., 2009; Bridger et al., 2013) and unsafe work behaviors (Wallace and Vodanovich, 2003; Day et al., 2012). Nevertheless, researchers of accidents have argued that cognitive failure represents only a fraction of the possible varieties of behavioral antecedents of accidents. They called for a shift to a broader approach which encompasses various categories of behavioral failures that should be treated separately in analysis (Reason, 1990; Lawton and Parker, 1998; Alper and Karsh, 2009) and might require different modes of remediation (Reason, 1990).

Such a multi-perspective approach has proven useful in the research of traffic accidents. A broad research program established that particular categories of driving behaviors serve as distinct pathways to accidents (e.g., Reason et al., 1990; Parker et al., 1995a). This research program used the Driver Behavior Questionnaire (DBQ; Reason, 1990): a self-report measure for aberrant driving behaviors. Reason et al. (1990) first study revealed a three-factor structure of the DBQ: Lapses, Mistakes, and Violations. The main distinction presented by this factor structure differentiates between errors that are unintended and violations that are deliberate deviations from prescribed rules and instructions. It also distinguished between two kinds of errors: mistakes, which are defined as “the departure of planned actions from some satisfactory path toward the desired goal” (mostly due to failures of judgment, estimation, and decision), and slips and lapses, which are defined as “the unwitting deviation of action from intention” (mostly due to failures of perception, attention or memory). Many studies that have investigated the factor structure of the DBQ have generally replicated the distinction between errors and violations, and their respective contributions to road accidents (Reason et al., 1990; Parker et al., 1995a; Beus et al., 2015). These patterns emerge more clearly from group (rather than individual) analyses, in which age and gender differences are the focus of evaluation. Each type of behavior was found to have different demographic correlates. Violations declined with age, while the relations between age and errors were found to be inconsistent. Men of all ages reported more violations than women. Women, however, were significantly more prone to harmless lapses (Reason et al., 1990; Parker et al., 1995a; Blockey and Hartley, 1995; Mecacci and Righi, 2006; de Winter and Dodou, 2010; Könen and Karbach, 2020). Some of those studies reproduced the three-factor structure of the DBQ, others (e.g., Åberg and Rimmö, 1998; Xie and Parker, 2002) obtained additional factors, and in some studies the content of the factors differed from those of Reason et al. (1990). The DBQ has been used extensively in the analysis of traffic accidents and in a few additional domains, such as aviation (Wiegmann and Shappell, 2001) and rail transport (Free, 1994). It has provided empirical data on the relationship between different types of behavioral failures and accidents (Reason et al., 1995; Jonah, 1997), and added considerable knowledge to the literature.

This body of evidence stresses the need to combine both human error and violations within a single research program, and demonstrated that neither one is sufficient to establish a precise relationship between behavioral tendencies and accidents. Since the DBQ has mainly been implemented in the research of traffic accidents, a gap exists in the understanding of the nature, sources, and corollaries of the various categories of human failure in additional areas (Lawton and Parker, 1998). Hence, a multifaceted approach to accident investigation and prevention could provide a powerful vehicle to assist in the understanding of human failures and the contexts in which they occur (Lawton and Parker, 1998).

### Choice of a Criterion – Methodological Issues

A major methodological challenge in the research of accidents and accident proneness is the issue of establishing criterion validity. Several researchers call to shift the focus of these studies to the behavioral antecedents of accidents rather than actual accidents (e.g., Elander et al., 1993; Lawton and Parker, 1998; Ulleberg and Rundmo, 2003; Sümer et al., 2005; Constantinou et al., 2011). These researchers assert that accidents are inherently unreliable as a dependent measure. Accidents are relatively rare and are influenced by various external factors (such as, coworkers, distractions, and system failure). As a result, the same unsafe behavior may in one instance incur no negative consequences, yet in another instance result in a fatal accident. It was suggested that, when studying the influence of psychological factors on behavior, a more appropriate and reliable criterion is an aggregation of different behaviors across situations (Epstein, 1979; Ulleberg and Rundmo, 2003; Sümer et al., 2005). According to Lawton and Parker (1998, p. 667), “This change of focus has already happened to some extent in relation to driving accidents. It is well established that driving above the posted speed limit is predictive of road traffic accidents in the long run. However, any attempt to demonstrate a direct link between speeding as measured in a single study and the occurrence of accidents within that study is unlikely to meet with success. Most speeding goes unpunished by negative consequences. However, that does not mean that speeding is not important in accident causation. Therefore, much research is now dedicated to determining the attitudinal and motivational characteristics that are associated with this dangerous driving behavior.” A similar approach is evident in additional domains. For example, there is substantial evidence that a large-scale adoption of officially recommended Covid-19 precautionary measures (e.g., social distancing and improving personal hygiene) by individuals is a key factor for reducing the spread of the pandemic (e.g., Cowling et al., 2020). Hence, research in this domain focuses on the factors influencing the (in)compliance to the Covid-19 guidelines rather than on the outcome of infection with the virus. Often, these studies employ self-reports to measure behavior. There is evidence that the driver’s self-reported behaviors are correlated with observers’ ratings of driver’s behavior (West et al., 1993) and that self-reported accidents and objective road safety data correlate fairly strongly (de Winter et al., 2015). Based on this literature, the current research relies on self-reported behaviors as a criterion for accident proneness.

### The Current Research

The current research was undertaken as a preliminary step toward the development and validation of the FP questionnaire, a brief, multifaceted self-report scale that assesses individual differences in the predisposition for behavioral failures in everyday settings. The FP focuses on psychological characteristics (rather than situational factors). Based on past studies (e.g., Broadbent et al., 1982; Larson et al., 1997; Finomore et al., 2009; Bridger et al., 2013), we assume that the predisposition to behavioral failures is a largely stable trait-like construct that predicts involvement in accidents and unsafe work behaviors (Wallace and Vodanovich, 2003; Day et al., 2012). Our aim was to develop an instrument that: (a) encompasses distinct categories of behavioral failures that occur in a wide variety of mundane contexts, and (b) is concise enough to use in research without taxing the participants. In developing the measure, we followed the steps advocated by the psychometric literature (Hinkin, 1998). The development of the measure included a preliminary phase of conceiving an extensive initial pool of prospective items, and a secondary phase encompassing three studies. Study 1 identified and validated the factor-structure of FP *via* exploratory and confirmatory factor analyses, condensing it to reach a conclusive set of items. Study 2 corroborated the factor structure of the FP through an additional confirmatory factor analysis, and evaluated its construct validity by assessing its relationship with the Big-Five Inventory (BFI) of personality traits. Although there are several psychological constructs that are linked in the literature to unsafe behaviors and accidents, we chose to focus on the FFM personality traits (McCrae and Costa, 1987), since these traits are some of the most frequently identified individual differences predictors of accidents and unsafe behaviors (e.g., Lawton and Parker, 1998; Wallace and Vodanovich, 2003; Clarke and Robertson, 2005). Yet, safety researchers have asserted that personality does not predict accidents directly, but rather it is a distal factor influencing accident proneness indirectly, through behavioral tendencies (e.g., speed choice and drunk driving), which are considered a proximal factor, directly related to accident risk (Elander et al., 1993; Sümer, 2003; Ulleberg and Rundmo, 2003). They have called for the identification of behaviors that mediate the relationships between personality and accidents (Hansen, 1989; Beus et al., 2015). Finally, Study 3 examined the ability of the FP questionnaire to predict individual differences in a population of job applicants undergoing a screening process, and tested the criterion-related validity of the FP by assessing its ability to predict deviant behaviors. The studies employed samples from different populations.

#### Conceiving an Initial Pool of Prospective Scale Items

Reason’s (1990) typology provided a useful starting point for item generation. Our goal was to generate a large pool of items tapping lapses, mistakes and violations that occur in a wide variety of contexts in daily life, which we would then reduce to a scale of 15–20 items. We sought to develop a measure that is concise, and yet encompasses comprehensive coverage of distinct categories of behavioral failures. Hence, in each category of failures, we included items that together, represent a broad range of relevant behaviors (rather than highly correlated similar behaviors). We prioritized content breadth over internal consistency (see John and Soto, 2007 for a similar approach). An initial pool of 90 items was created by a team of three occupational psychologists and four occupational psychology graduate students, who independently listed behaviors believed to represent failures in everyday environments. The first author and an occupational psychology graduate student then reviewed the items and verified that all were clearly worded and relevant to a wide variety of contexts. They also eliminated redundant (overlapping) items and combined items with similar content. This process produced 45 items.

An additional step in the development of the FP involved obtaining preliminary feedback from a panel of ten occupational psychology students who were provided with definitions of lapses, mistakes, and violations, and were asked to classify each item in one of these three categories. Items sorted by fewer than 80% of the raters into an expected category were discarded from the scale (Wallace and Chen, 2005). This process yielded 30 items for the initial FP.

## Study 1

Study 1 was aimed at identifying the factor-structure of the FP questionnaire, and evaluating its validity in a large sample, *via* exploratory and confirmatory factor analysis. Another important objective of this study was to reduce the 30-item FP to a more abbreviated scale. Although the development of the FP was based on Reason’s (1990) typology, we conducted both exploratory and confirmatory factor analyses to accurately evaluate the FP’s dimensionality. Since Reason’s (1990) typology was employed mainly for the investigation of traffic accidents, and given the variability in the dimensional-structure identified by previous factor-analytic studies of the DBQ (e.g., Åberg and Rimmö, 1998), no assumptions were drawn regarding the number of factors of the FP. We expected to find at least three factors analogous to the categories proposed by Reason, but assumed that additional factors might also emerge.

### Method

#### Participants

Our recruitment efforts *via* social media, professional forums, and email yielded 586 volunteers. Of the participants, 18 were identified as multivariate outliers using the Mahalanobis D2 method (*p* < 0.001). The final sample included 568 adult participants (ages between 18 and 75, *M* = 34.43, *SD* = 11.94, 66.4% women). Of these, 427 (75.2%) participants had an academic degree.

Our goal was to recruit as many participants as possible to ensure stable factors. Kline (1991) declared that a factor analysis must include at least 100 participants, at least two participants per item and at least 20 participants per extracted factor. With 30 items and 3–7 factors expected to emerge, the sample met or exceeded all three criteria. Hence, this sample was sufficient for us to conduct a factor analysis with confidence in the results.

#### Measures

##### Failures Proneness Questionnaire

The FP developed in earlier stages comprised 30 items describing everyday behaviors and situations. The participants were asked to indicate how often they experience each of them, on a Likert scale, ranging from 1 (never), to 7 (very often).

#### Procedure

The study was conducted online using Google Forms. Informed consent was obtained before data collection. All participants completed the FP in a single session. Age and gender were also indicated.

#### Statistical Approach

Participants were randomly divided into two groups: one for an initial exploratory factor analysis (EFA), and the second for a confirmatory factor analysis (CFA) and additional item-reduction to improve fit (“pruning”). The CFA in this study was therefore utilized as an additional exploratory measurement.

##### Exploratory Factor Analysis

Principal axis factoring was conducted on 30 items using oblique rotation, constrained to a maximum of 25 rotation iterations. Oblique rotation methods allow for the more realistic underlying assumptions of inter-factor correlations. The number of factors was determined by examining the results of several methods, including scree plot, Kaiser rule (number of eigenvalues 1), and theoretical consideration (i.e., expectation that different factors would emerge for different hypothesized dimensions). In subsequent analyses this was constrained to 4–7 factor solutions. Items with individual loadings below 0.4, cross-loadings above 0.3, communality below 0.2, or content inconsistent with other items in its factor were eliminated (Howard, 2016).

##### Confirmatory Factor Analysis

We used a maximum likelihood CFA on the remaining items from the EFA. As part of the CFA procedure, we used “goodness of fit” criteria of χ^2^/df ratio of <3.0, goodness of fit index (GFI) > 0.95, a non-normed fit index (“Tucker Lewis Index”; TLI) > 0.95, a comparative fit index (CFI) of 0.95 or greater, root-mean-square error of approximation (RMSEA) of <0.05 (Bentler and Bonett, 1980; Carmines and McIver, 1981; Schermelleh-Engel et al., 2003; Iacobucci, 2010), and standardized root mean square residual (SRMR). Based on the magnitude of cross loadings with other factors, additional items were removed, to ensure that all modification indices had values that met each of the decision rules. Potential limitations of these indices include sensitivity to model misspecification, small sample bias, estimation method effect, effects of violation of normality and independence, and bias of fit indices resulting from model complexity (Schermelleh-Engel et al., 2003). After CFA, we ran an additional EFA on the EFA sample with the final version of the FP in order to validate the CFA solution.

### Results and Discussion

#### Exploratory Factor Analysis

A Principal Axis Factoring (PAF) with oblimin rotation performed on the first half of the sample (N1 = 284Ss) yielded (in 13 iterations) six factors, with a KMO measure of sampling adequacy of (0.80) and a good Bartlett’s test of Sphericity [χ^2^(435) = 2697.39, *p* < 0.001], which explained 51.24% of the total variance (pre-extraction) and a total of 40.99% of the variance (post-extraction). Seven items were excluded (because of low individual loadings, high cross-loadings, or content inconsistent with other items in the factor) and the EFA was re-run. The 23-item solution was achieved in nine iterations and yielded a KMO measure of sampling adequacy of (0.79), and a good Bartlett’s test of Sphericity [χ^2^(253) = 1879.09, *p* < 0.001]. The 23-item solution explained 57.89% of the total variance (pre-extraction) and a total of 44.45% of the variance (post-extraction). The six-factor structure was maintained perfectly regarding the item inclusion criteria, with sufficient loadings and no cross-loadings. Communalities of the variables ranged between 0.20 and 0.85.

#### Confirmatory Factor Analysis

**FIGURE 1:**
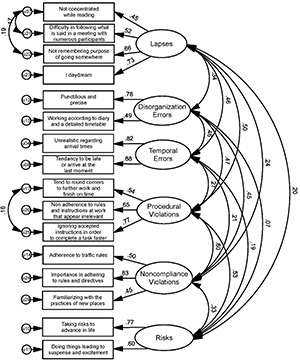
Factor loadings using the 16-item final FP solution and correlations between FP subscales.

The 23-item six-factor EFA solution was then modeled using the AMOS program. A Maximum Likelihood CFA procedure executed on the second half of the randomly split sample (N2 = 284Ss) did not yield satisfactory fit indices. Therefore, using some of the suggested modification indices to reduce cross loadings, seven items were removed, and to account for some within-factor non-zero correlations between unobserved error variances, some correlation arcs were added to the unobserved error measures. The final six-factor model had 16 items with the following fit statistics: χ^2^/df = 1.341 (χ^2^ = 115.32, df = 86, *p* = 0.02), GFI = 0.95, TLI = 0.96, CFI = 0.97, RMSEA = 0.035 (CI90 = [0.015, 0.05], *p*(RMSEA) < 0.05 = 0.95) and SRMR = 0.042. These indices represent a good fit of the model based on the reported criteria. Since the CFA led to further elimination of items, an additional EFA was performed on the original EFA dataset, to validate the final 16-item FP. This EFA perfectly replicated the factor structure of the CFA. The solution explained 67.31% of the total variance (pre-extraction) and a total of 49.64% of the variance (post-extraction). The factor loadings of the 16-item FP and the correlations in the CFA sample are presented in Figure 1. Cronbach’s alpha reliability coefficients for both EFA and CFA samples are presented in Table 1.

**TABLE 1:** Cronbach’s α Reliabilities, means and standard deviations of FP scales – as obtained in the two samples in Study 1.

	** CFA sample (*N* = 284)**		** EFA sample (*N* = 284)**	
	***α***	***M (SD)***	***α***	***M (SD)***
Lapses (LP)	0.72	3.53(1.16)	0.71	3.65(1.12)
Disorganization-errors (DE)	0.54	2.95(1.27)	0.57	3.21(1.36)
Temporal-errors (TE)	0.84	3.22(1.75)	0.86	3.19(1.70)
Procedural-violations (PV)	0.70	3.13(1.15)	0.70	3.27(1.16)
Non-compliance-violations (NV)	0.61	2.73(1.05)	0.66	2.87(1.08)
Risks (RK)	0.63	3.53(1.27)	0.65	3.52(1.25)
FP general	0.79	3.19(0.75)	0.80	3.30(0.77)

In summary, several rounds of exploratory and confirmatory factor analyses yielded a 16-item questionnaire. The structure that emerged in our data comprises six distinct factors: The first, “Lapses” (four items), resembles Reason’s notion of lapses and Broadbent et al.’s (1982) notion of cognitive failure. It captures failures in attention, alertness, memory and daydreaming, which represent off-task behaviors that could hinder performance (Wallace and Chen, 2005). The second and third factors represent failures in estimation and judgment, and thus are congruent with Reason’s definition of mistakes. These are “Disorganization-Errors” that denote general disorganization, and “Temporal-Errors” that depict maladaptive time management, which are related to adverse negative consequences, such as inadequate job performance, lower wage (Metin et al., 2018) and impaired academic performance (Macan and Shahani, 1990). The fourth and fifth factors denote deliberate deviation from prescribed rules and hence parallel Reason’s definition of violations. These are “Procedural-Violations,” which describe deviance from guidelines or regulations in order to promote other goals perceived as more valuable, and “Non-compliance-Violations” associated with non-conforming attitudes and low internalization of norms. Violations are widespread in various occupational sectors (McKeon et al., 2006) and are linked to accidents and exceptional safety incidents (Parker et al., 1995b; Dekker, 2002). The distinction between different types of violations is common in the literature (Reason, 1990). The sixth factor – “Risks” – is conceptually similar to sensation and risk seeking (Zuckerman, 2015), which is related to delinquency (Ljubin-Golub et al., 2017) and to involvement in accidents (Zhang et al., 2019). Our data is congruent with Reason’s (1990) original theoretical distinction between lapses, mistakes, and violations, and expands it to everyday environments. Moreover, it also distinguishes between different types of mistakes and between different kinds of violations, and hence may be more informative for theoretical and practical purposes.

Notably, the correlations between the six factors and the general factor of FP are medium-high. This might suggest that despite being distinct factors, there is a common thread between the different types of failures, which contribute to a general factor of failures tendency.

#### Demographic Correlates

Several studies have reported that different types of behavioral failures have different demographic correlates, with men reporting more violations than women while women describing more lapses. Additionally, violations (but not lapses) tended to decrease with age (Parker et al., 1995a; de Winter and Dodou, 2010). Hence, we performed independent samples t-tests to examine whether similar gender-differences and age correlates appeared in our data (Table 2).

**TABLE 2:** Means and standard deviations of the FP scores for men and women and their correlation with age.

	**Gender**						**Age**
	**Female (*n* = 377)**		**Male (*n* = 191)**				
	***M***	***SD***	***M***	***SD***	***t(566)***	***r(pb)***	***r***
Lapses	3.66	1.15	3.45	1.12	−2.06*	−0.09*	−0.27**
DE	2.91	1.30	3.42	1.30	0.44***	0.14**	−0.16**
TE	3.22	1.75	3.17	1.68	–0.30	–0.01	−0.17**
PV	3.06	1.09	3.48	1.23	4.10***	0.14**	−0.11**
NV	2.70	1.04	3.00	1.08	3.36***	0.22**	−0.21**
Risks	3.38	1.22	3.80	1.29	3.78***	0.18**	−0.17**
FP general	3.18	0.77	3.38	0.73	2.90***	0.14**	−0.30**

*p < 0.05, **p < 0.01, ***p < 0.001.

As Table 2 shows, men scored significantly higher on Procedural-Violations, Non-compliance and Risks, while women scored significantly higher on Lapses. In addition, men reported significantly more Disorganization-Errors and had a higher FP total score. Gender differences on Temporal Errors were not significant. Pearson correlations revealed that scores of all categories of behavioral failures declined with age. These gender differences and associations with age are congruent with previous research (Reason et al., 1990; Parker et al., 1995a; de Winter and Dodou, 2010), and hence strengthen the structure validity of the FP’s factor structure.

## Study 2

The goal of Study 2 was twofold: (a) to retest the factor structure of the FP *via* an additional confirmatory factor analysis in an independent sample, and (b) to evaluate its convergent and divergent validity by assessing its relationship with the FFM personality traits of Conscientiousness, Agreeableness, Neuroticism, Extraversion and Openness to experience (McCrae and Costa, 1987). FFM personality traits are linked to distinct sets of motivations and behaviors (Barrick et al., 2013) and are some of the most frequently identified individual difference predictors of accidents and unsafe behaviors (Lawton and Parker, 1998; Wallace and Vodanovich, 2003; Bogg and Roberts, 2004; Clarke and Robertson, 2005; Beus et al., 2015). Yet, research on the association between personality traits and accident involvement has often produced weak correlations (Herzberg, 2009). Consequently, researchers have called for the identification of behaviors that mediate the relationships between personality and accidents (e.g., Hansen, 1989). In this respect, behavioral failures were highlighted as a powerful mediator of these associations (Wallace and Vodanovich, 2003; Beus et al., 2015).

*Conscientiousness* is characterized by responsibility, efficiency, organization, and rule-compliance (McCrae and Costa, 1987; Barrick et al., 2013), and is negatively correlated with unsafe behaviors (Wallace and Vodanovich, 2003; Beus et al., 2015). Hence, we expect a negative association between conscientiousness and FP scores, and posit that this relationship will be manifested in all the FP’s factors.

*Agreeableness* is characterized by cooperation, trust, and compliance (McCrae and Costa, 1987). It is negatively related to unsafe behaviors and accidents (Cellar et al., 2001; Clarke and Robertson, 2005; Sümer et al., 2005; Beus et al., 2015). Thus, we predict that Agreeableness will be negatively associated with the overall FP score, in particular to Risks, Procedural-Violations, and Non-compliance-Violations.

*Neuroticism* is linked to anxiety, stress, preoccupation with negative emotions (McCrae and Costa, 1987), distracted thinking (Buchanan, 2016), and risk avoidance (Nicholson et al., 2005). Several studies have indicated that Neuroticism is positively associated with unsafe behaviors and accidents (Neal and Griffin, 2006; Christian et al., 2009; Beus et al., 2015), while others found a negligible relationship (Clarke and Robertson, 2005) or no relationship (O’Hern et al., 2020). This inconsistency could imply that context moderates this relationship (Lajunen, 2001; Clarke and Robertson, 2005). We postulate that Neuroticism will be positively associated with the overall FP score, especially with Lapses, Disorganization-Errors, and Temporal-Errors, but will be negatively correlated with Risks.

*Extraversion* is characterized by sociability, dominance (McCrae and Costa, 1987), and influence motivations (Barrick et al., 2013). It is associated with sensation-seeking and lower vigilance (Koelega, 1992; Eysenck, 2013), as well as positive affect (Eysenck, 2013), high self-evaluations (Judge et al., 2003), and better attentional focus (Hahn et al., 2015). Consequently, the literature presents a complex picture of the relationships between Extraversion and accidents (Clarke and Robertson, 2005). Several empirical studies have presented positive correlations (Lajunen, 2001; O’Hern et al., 2020), while others found the opposite effect (e.g., Pestonjee and Singh, 1980) or no correlation (e.g., Clarke and Robertson, 2005; Christian et al., 2009). Additionally, several studies found a negative association between Extraversion and cognitive failure (Könen and Karbach, 2020; Sutin et al., 2020) whereas others yielded no relationship (Wallace, 2004). Accordingly, we expect Extraversion to be positively related to Risks, and have no prediction for its relationship with Lapses and Disorganization-Errors and Temporal-Errors.

*Openness* is associated with cognitive flexibility, preference for variety, intellectual curiosity (McCrae and Costa, 1987), autonomy-seeking (Barrick et al., 2013) questioning of authority, dissatisfaction with routine (Smith et al., 2018), and proneness to deviant behaviors (Salgado, 2002). The occupational safety literature contains few studies of Openness (Christian et al., 2009). Clarke and Robertson (2005) found it to be positively related to accident-involvement, but suggested that this relationship is moderated. Other studies found no relationship (Christian et al., 2009; Beus et al., 2015; O’Hern et al., 2020). The association of the CFQ to Openness appeared minor and unstable (Snitz et al., 2015; Könen and Karbach, 2020; Sutin et al., 2020). Based on the inconclusive findings in previous studies, we hypothesize that Openness will be positively correlated with Risks, Procedural-Violations, and Non-compliance, and posit no hypothesis regarding its relationship to Lapses, Disorganization-Errors, and Temporal-Errors.

### Method

#### Participants

A total sample of 692 participants was composed of 555 undergraduate psychology students from a university in central Israel (participating in the study as part of their academic requirements), and 137 volunteers who were recruited through social media, professional forums, and by email. Of the 692 adult participants who completed the questionnaire, 28 were identified as multivariate outliers using the Mahalanobis D2 method (*p* < 0.001). The final sample included 664 adult participants [ages between 18 and 69, *M* = 29.88, *SD* = 9.10, 74.7% women (*n* = 496), 25.0% men (*n* = 166), 0.3% gender not specified (*n* = 2)].

#### Measures

##### Failures Proneness Questionnaire

We used the 16-item FP finalized in Study 1.

Table 3 presents the reliabilities of the FP in Study 2, compared to the reliabilities found in the two Study 1 samples, showing a similar and consistent pattern of reliability coefficients.

**TABLE 3:** Cronbach’s α reliabilities, means, and standard deviations of the FP scores – Study 2 in comparison to Study 1.

	**Study 2 sample (*N* = 664)**		**Study 1 samples (both *N*’s = 284)**	
	***α***	***M (SD)***	***α (EFA)***	***α (CFA)***
LP	0.63	3.85(1.15)	0.71	0.72
DE	0.47	2.94(1.28)	0.57	0.54
TE	0.77	3.08(1.62)	0.86	0.84
PV	0.67	3.33(1.30)	0.70	0.70
NV	0.61	2.74(1.10)	0.66	0.61
RK	0.66	4.15(1.43)	0.65	0.63
FP general	0.77	3.19(0.75)	0.80	0.79

##### Personality

The Hebrew short version (Etzion and Laski, 1998) of the 44-item Big Five Inventory (BFI; John et al., 2008) of personality traits (McCrae and Costa, 1987) was used. The BFI consists of 44 items that are rated by participants on a five-point Likert scale ranging from 1 (strongly disagree) to 5 (strongly agree) and measures five broad personality traits, including Conscientiousness (9 items, α = 0.74), Agreeableness (9 items, α = 0.75), Neuroticism (8 items, α = 0.80), Extraversion (8 items, α = 0.78), and Openness (10 items, α = 0.76), along with two narrower facets within each trait (Soto and John, 2017).

#### Procedure

The study was conducted online using Qualtrics. Informed consent was obtained before data collection. Participants first completed the FP, and then the BFI. Age and gender were also indicated. All questionnaires were administered in a single session.

### Results and Discussion

#### Descriptive Statistics

Table 4 presents descriptive statistics and consistencies for all variables measured in Study 2. Table 5 presents descriptive statistics segmented by gender.

**TABLE 4:** Descriptive statistics of study 2’s variables.

	***M***	***SD***	**Min**	**Max**
B5 conscientiousness	3.93	0.57	1.56	5.00
*B5 order (C)*	3.62	0.96	1.00	5.00
*B5 self discipline (C)*	3.86	0.64	1.40	5.00
B5 agreeableness	3.94	0.60	1.56	5.00
*B5 altruism (A)*	4.02	0.65	1.50	5.00
*B5 compliance (A)*	3.82	0.74	1.33	5.00
B5 neuroticism	2.80	0.75	1.00	5.00
*B5 anxiety (N)*	2.78	0.83	1.00	5.00
*B5 depression (N)*	2.68	1.03	1.00	5.00
B5 extraversion	3.44	0.70	1.50	5.00
*B5 assertiveness (E)*	3.14	0.82	1.20	5.00
*B5 activity (E)*	3.80	0.85	1.00	5.00
B5 openness	3.77	0.60	2.00	5.00
B5 aesthetics (O)	3.58	1.00	1.00	5.00
B5 ideas (O)	3.75	0.60	2.00	5.00
Lapses	3.85	1.15	1.00	7.00
DE	2.94	1.28	1.00	7.00
TE	3.08	1.62	1.00	7.00
PV	3.33	1.30	1.00	6.67
NV	2.74	1.10	1.00	7.00
Risks	4.15	1.43	1.00	7.00
FP general	3.37	0.77	1.13	5.94

**TABLE 5:** Means and standard deviations of the FP scores for men and women and their correlation with age.

	**Gender**						**Age**
	**Female (*n* = 496)**		**Male (*n* = 166)**				
	***M***	***SD***	***M***	***SD***	***t(660)***	***r(pb)***	***r***
B5 conscientiousness	3.98	0.57	3.80	0.56	−3.41***	−0.13**	0.15**
*B5 order (C)*	3.70	0.97	3.39	0.92	−3.59***	−0.14**	0.05
*B5 self discipline (C)*	3.89	0.64	3.76	0.62	−2.34*	−0.09*	0.17**
B5 agreeableness	3.98	0.60	3.84	0.57	−2.69**	−0.10**	0.08*
*B5 altruism (A)*	4.06	0.65	3.90	0.65	−2.82**	−0.11**	0.08*
*B5 compliance (A)*	3.85	0.74	3.71	0.70	−2.13*	−0.08*	0.00
B5 neuroticism	2.87	0.73	2.61	0.77	−3.82***	−0.15**	−0.12**
*B5 anxiety (N)*	2.86	0.82	2.52	0.80	−4.63***	−0.18**	–0.07
*B5 depression (N)*	2.72	1.04	2.58	1.01	1.47	–0.06	−0.15**
B5 extraversion	3.48	0.70	3.32	0.70	−2.62**	−0.10**	0.02
*B5 assertiveness (E)*	3.19	0.82	3.00	0.80	−2.59*	−0.10**	0.01
*B5 activity (E)*	3.83	0.86	3.73	0.83	–1.33	–0.05	0.02
B5 openness	3.74	0.59	3.85	0.64	2.13*	0.08*	0.02
B5 aesthetics (O)	3.61	0.98	3.50	1.08	–1.19	–0.05	0.04
B5 ideas (O)	3.68	0.58	3.94	0.64	4.94***	0.19**	0.00
Lapses	3.91	1.18	3.68	1.04	−2.27*	−0.09*	−0.16**
DE	2.84	1.28	3.25	1.24	3.60***	0.14*	–0.03
TE	3.07	1.62	3.12	1.64	0.38	0.01	−0.11**
PV	3.22	1.33	3.63	1.17	3.60***	0.14**	–0.04
NV	2.60	1.07	3.14	1.09	5.66***	0.22**	–0.07
Risks	4.01	1.42	4.59	1.36	4.67***	0.18**	−0.12**
FP general	3.31	0.78	3.56	0.70	3.71***	0.14**	−0.16**

*p < .05, **p < .01, ***p < .001.

To explore the gender differences in behavioral failures, we ran independent samples *t*-tests. Point-by-serial correlations were used for effect power. As Table 5 shows, men scored significantly higher on Procedural Violations, Non-compliance Violations and Risks, while women scored significantly higher on Lapses. In addition, men reported significantly more Disorganization Errors and had a higher FP total score. Gender differences on Temporal Errors were not significant. Pearson correlations revealed that scores of Lapses, Temporal Errors, Risks, and FP general declined with age.

##### Confirmatory Factor Analysis

A confirmatory factor analysis with the fit criteria described in Study 1 yielded an identical 16-item solution with similar factor loadings and similar fit indices: χ^2^/df = 2.217 (χ^2^ = 190.69, df = 86, *p* < 0.001), GFI = 0.97, TLI = 0.93, CFI = 0.95, RMSEA = 0.043 (CI90 = [0.035, 0.051], *p*(RMSEA) < 0.05 = 0.92) and SRMR = 0.037. These indices represent a good fit of the model, based on the reported criteria, and thus provided a constructive replication of Study 1.

##### Correlations With the Big-Five Inventory

In order to explore the relationship between personality and behavioral failures, we calculated the Pearson correlations between the FP and the Big-Five personality traits and facets (Table 6).

**TABLE 6:** Correlations among BFI (traits and facets) and FP scales.

	**LP**	**DE**	**TE**	**PV**	**NV**	**RK**	**FP general**
Conscientiousness	−0.44**	−0.57**	−0.31**	−0.44**	−0.36**	−0.09*	−0.62**
C-order	−0.25**	−0.44**	−0.25**	−0.35**	−0.25**	−0.11**	−0.46**
C-discipline	−0.48**	−0.51**	−0.28**	−0.39**	−0.33**	–0.06	−0.59**
Agreeableness	−0.14**	−0.15**	−0.12**	−0.24**	−0.23**	–0.04	−0.26**
A-altruism	−0.15**	−0.18**	−0.11**	−0.22**	−0.22**	–0.01	−0.25**
A-compliance	–0.06	–0.07	−0.08*	−0.17**	−0.13**	–0.04	−0.16**
Neuroticism	0.36**	0.08*	0.10**	0.09*	0.03	−0.11**	0.19**
N-anxiety	0.31**	0.04	0.05	0.01	–0.03	−0.15**	0.10*
N-depression	0.30**	0.12**	0.13**	0.19**	0.07	0.03	0.25**
Extraversion	−0.18**	−0.12**	–0.04	−0.08*	0.02	0.18**	−0.08*
E-assertiveness	−0.15**	−0.08*	–0.03	–0.05	0.07	0.14**	–0.04
E-activity	−0.16**	−0.14**	–0.04	−0.11**	−0.08*	0.19**	−0.11**
Openness	0	–0.01	0.08*	0.06	0.03	0.22**	0.10*
O-aesthetics	–0.02	–0.06	0.03	0.02	–0.05	0.07	0
O-ideas	0.04	0.05	0.12**	0.10**	0.14**	0.27**	0.19**

*p < 0.05, **p < 0.01.

In line with our hypotheses, convergent and divergent relationships with the BFI were found. The predisposition for behavioral failures (as indicated by an overall FP score) was negatively related to both Conscientiousness (−0.62) and Agreeableness (−0.26), and positively related to Neuroticism (0.19), suggesting that individuals higher in Conscientiousness and Agreeableness and lower in Neuroticism are less prone to behavioral failures. These findings are consistent with ample evidence on the relationship between personality and cognitive failures (Wallace, 2004; Aschwanden et al., 2020; Könen and Karbach, 2020), and safety behaviors and accidents (Lawton and Parker, 1998; Clarke and Robertson, 2005; Beus et al., 2015). In regard to divergent relationships, the low to medium correlations of the FP with Agreeableness, Openness, Neuroticism and Extraversion lends support to the separability of FP and BFI.

A closer scrutinization of the relationship between each of the five personality traits with the first-order factors of the FP highlights several interesting patterns, which could reflect a joint operation of the narrower facets (Soto and John, 2017).

Conscientiousness was negatively correlated to all FP factors. These associations apparently reflect the broad nature of Conscientiousness (Wallace and Vodanovich, 2003; Barrick et al., 2013). Additionally, both lower-order facets of Conscientiousness, Order and Self-Discipline were negatively associated with all these factors. These patterns suggest that the lesser tendency of individuals high in Conscientiousness to commit behavioral failures is due both to their sense of organization and their tendency to adhere to norms and rules. Agreeableness was negatively associated with all FP factors except Risks. While the violation factors were associated with both Altruism and Compliance, Lapses, Disorganization-Errors, and Temporal-Errors were only associated with altruism.

The weak correlation of Neuroticism with the FP general score appears to be the outcome of its contrasting patterns of correlations with the FP’s factors, with a positive correlation to Lapses, Disorganization-Errors, Temporal-Errors and Procedural-Violations and a negative correlation with Risks. These correlations generally follow the expected patterns described in the literature (e.g., Hohman et al., 2011; Sutin et al., 2020). The positive association with Lapses appears for both Anxiety and Depression facets and hence might be due to worry and lack of energy which functions as a form of cognitive distraction (Denovan et al., 2019), and as a higher inclination toward engaging in task-irrelevant thoughts (Judge et al., 2003). The positive correlations with Temporal-Errors, Disorganization-Errors, and Procedural-Violations are evident only for the Depression facet, and therefore might imply that the lack of energy associated with Depression (Milanovic et al., 2018) encourages the use of shortcuts in order to minimize effort. The negative correlation with Risks is reflected in the Anxiety facet, and hence could reflect risk avoidance (Nicholson et al., 2005).

Although Extraversion was unrelated to the total FP score, it had differing correlations across the FP factors, with positive correlations for Risks, and negative correlations for Lapses and Disorganization-Errors. These contradictory patterns, reflected both in the Activity and in the Assertiveness facets of Extraversion, highlight the multifaceted relationship of Extraversion with behavioral failures (Christian et al., 2009).

Although there were no hypotheses for Openness, a positive correlation emerged for Risks (0.22). This correlation corresponds with the description of individuals who rate high in their Openness to adventure and daring (McCrae and Costa, 1987). The lack of correlations with the other FP factors is apparently related to a lack of correlation with the Aesthetic facet. In fact, when focusing on the lower facet of Ideas, additional significant correlations emerged with both Procedural-Violations, Non-compliance, and Disorganization-Errors. These patterns might reflect the higher tendency of individuals rating high in ideas to breach regulations.

## Study 3

Tools for predicting behavior are critical for those making selection and promotion decisions in organizational contexts (Cohen et al., 2014). As organizations across the globe become aware of the costs incurred from behavioral failures and accidents, they are increasingly looking for diagnostic tools for evaluating employee performance and making personnel selections (Casillas et al., 2009). Selective hiring offers a proactive approach to maintaining work place safety (Cohen et al., 2014). Yet, most procedures used to predict safety performance have typically focused solely on personality characteristics (MacLane and Walmsley, 2010) and there are calls for research aimed at identifying important individual difference predictors of safety performance to advance selection procedures and promote safer work environments (Cunningham et al., 2018). Consequently, a primary objective of Study 3 is to examine the ability of FP to predict individual differences and to substantiate the reliability of its factors in a population of job applicants undergoing a screening process. Another objective of this study was to further validate the FP by assessing its relationship with predisposition to deviant behaviors, as measured *via* an integrity questionnaire. Integrity inventories prevail in personnel selection systems and are considered to be criterion-focused scales (Ones and Viswesvaran, 2001). Overt integrity questionnaires, which pose direct questions on the frequency of past deviant behaviors (e.g., alcohol consumption and truthful reporting) and on attitudes toward such behaviors, were identified as strong predictors of broad counterproductive work behaviors such as rule-breaking, accident involvement, and property damage (Ones and Viswesvaran, 2001), and of deviant behaviors outside the workplace (Lucas and Friedrich, 2005).

In this study, we heeded Lawton and Parker’s (1998) suggestion to focus on behavioral failures as precursors of accidents. Whereas accident rates are inherently unreliable as a dependent measure (Lawton and Parker, 1998), deviant behaviors that are widespread in various contexts increase accident probability (Jones, 1991). Deliberate violations of norms and non-adherence to expected behavior were highlighted also by Reason (1990) as antecedents of accidents. We postulate a positive association between FP scores (mainly the violations and risk factors) and deviant behaviors.

### Methods

#### Participants

Participants included 187 adults who applied for a variety of administrative, manufacturing, engineering, and project manager positions in a large Israeli electrical appliance corporation and were in the process of pre-employment screening. Of these, 16 participants were identified as multivariate outliers using the Mahalanobis D2 method (*p* < 0.001). The final sample included 171 adults (52.0% males). Statistics relating to age are not available, as Israeli law forbids employers to query the age of applicants during screening.

#### Measures

##### Integrity Questionnaire

A self-reporting integrity questionnaire developed by Psiphas Psychological Applications Ltd was used^1^. It consists of questions relating to distinct subscales of deviant behaviors and attitudes. These include integrity deviations (e.g., “Has a lawsuit ever been filed against you in court?”), commitment to the organization (e.g., “were you ever reprimanded or scolded for inappropriate behavior in the workplace?”), property offenses (e.g., “I once received money or goods from someone in a dishonest way”), truthful reporting (e.g., “On several occasions I argued with my superior at work and later regretted it”), drug abuse (e.g., “In the last 5 years, substance usage impaired my functioning at work at least once”), bribery (e.g., “I did not tell my superiors about the bribe I was offered”), alcohol consumption (e.g., “I drove under the influence of alcohol at least once during the last 5 years), gambling (e.g., “have you ever gambled a sum that was greater than half of your monthly income?”), and violence (e.g., “I tend to carry a knife with me for self-defense”). The test contains 250 items. Participants were instructed to answer “yes” or “no” to each item. Integrity scores on each subscale consisted of the sum of “yes” answers. Cronbach’s α reliability for integrity subscales is between 0.51 (violence) and 0.79 (drug abuse).

##### Failures Proneness Questionnaire

The same FP scale as administered in Studies 1 and 2.

#### Procedure

Data collection was conducted courtesy of, and in collaboration, with Psiphas Psychological Applications Ltd. The FP questionnaire was incorporated into a battery of tests and questionnaires (including the integrity questionnaires used in this study) completed by job applicants as part of a screening process.

### Results and Discussion

#### Descriptive Statistics

The FP mean scores and reliability data obtained in Study 3 are presented in Table 7.

**TABLE 7:** Means, standard deviations and Cronbach-α reliabilities of FP subscale, as obtained in Study 3.

**FP sub scales**	***M* (*SD*)**	**α**
LP	2.26(0.75)	0.65
DE	1.98(1.08)	0.56
TE	1.89(0.89)	0.76
PV	1.80(0.82)	0.66
NV	1.74(0.76)	0.72
RK	2.84(1.43)	0.70
FP general	2.07(0.63)	0.84

As Table 7 shows, the reliabilities of the FP scale obtained in study 3 are similar to those obtained in studies 1 and 2. The FP mean scores in this study were lower and close to the lower boundary of the response scale. This pattern is probably due to the tendency of job applicants to under-report undesirable behaviors. Despite this tendency, significant interpersonal variability in deviant behavior and significant relationships with the FP were obtained.

#### Correlations Between Failures Proneness Questionnaire Subscales and Integrity Scales

The Pearson correlations between the FP factors and the Integrity scales are presented in Table 8.

**TABLE 8:** Correlations between integrity scales and FP scales.

	**LP**	**DE**	**TE**	**PV**	**NV**	**RK**	**FP general**
Alcohol consumption	0.15*	0.10	0.13	0.22**	0.12	0.29**	0.25**
Bribery	0.20**	0.17*	0.16*	0.33**	0.23**	0.16*	0.31**
Truthful reporting	0.35**	0.26**	0.34**	0.40**	0.31**	0.29**	0.47**
Drug abuse	0.20**	0.19*	0.16*	0.35**	0.29**	0.41**	0.41**
Gambling	0.18*	0.20**	0.29**	0.31**	0.28**	0.22**	0.35**
Commitment to organization	0.42**	0.31**	0.40**	0.57**	0.42**	0.36**	0.59**
Property offense	0.35**	0.33**	0.22**	0.44**	0.33**	0.38**	0.50**
Violence	0.16*	0.07	–0.02	0.19*	0.16*	0.01	0.16*
Integrity deviations	0.07	0.12	0.23**	0.19*	0.15*	0.09	0.20**

*p < 0.05, **p < 0.01.

In accordance with our assumption, the Integrity scales were positively and significantly correlated to the FP general scores. These relationships were reflected in the bivariate correlations among the first-order FP factors and all Integrity scales, which were weak-to-moderate and mostly significant. Higher FP scores were related to deviant behaviors such as alcohol consumption (0.25), bribery (0.31), truthful reporting (0.47), drug abuse (0.41), gambling (0.35), deviations in organizational commitment (0.59), property offenses (0.50), and violence (0.16). In support of our hypothesis, factors in the FP were positively associated with most deviant behaviors. This was noted more in Procedural Violations than in Non-compliance-Violations. Procedural-Violations include an element of discretion and decision-making rather than blatant disregard of rules, and the current findings point to the importance of this element in understanding behavioral failures. Lapses and errors factors were also weakly and moderately positively associated with the various integrity scales (all significant *r*’s between 0.15 and 0.42). This suggests the existence of a general construct underlying FP and deviant behaviors.

In conclusion, Study 3 served to examine psychometric qualities of the FP among job applicants in the process of pre-employment screening and to further examine its validity related to deviant behaviors. In accordance with the findings of Study 1, satisfactory quality psychometrics were found. However, it appeared that a certain degree of social desirability bias may have contributed to lower FP means as compared to previous studies. Noteworthy for all the deviant behaviors examined in this study, the FP general score predictive ability was similar or higher than that of the specific factors. This pattern implies that the overall behavioral failure construct, as a higher order factor, captures the processes underlying deviant behaviors.

## General Discussion

The current research was undertaken as a first step in conceptualizing behavioral failures proneness in its multidimensional aspect. We report the initial stage of developing and validating the Failures Proneness questionnaire, an all-encompassing measure of common behavioral failures in daily life. The newly formulated questionnaire heeds Lawton and Parker’s (1998) call for a multifaceted research approach, which incorporates distinct categories of behavioral antecedents of accidents (Lawton and Parker, 1998). It includes six reliable and valid factors which encompass both cognitive components implicated by human errors (Lapses, Disorganization-Errors, and Temporal-Errors), and motivational components associated with deliberate violations and risky behaviors (Procedural-Violations, Non-compliance-Violations, and Risks). This factor structure is congruent with Reason’s (1990) typology of lapses, mistakes and violations, while also offering a distinction between different types of mistakes and different types of violations.

Our data confirmed that the 16-item factor structure of FP is reliable across studies and populations. Several rounds of exploratory and confirmatory factor analyses with different populations consistently yielded the same solution with similar factor loadings and similar fit indices. Furthermore, Construct validity was gained, as all the expected patterns of association with the FFM personality traits were obtained and reached significance. Criterion validity of FP was gained, as FP was significantly related to a wide range of deviant behaviors.

The divergent relationships obtained in Study 2 between the FFM personality traits and the distinct categories of behavioral failures highlights the multifaceted nature of the FP and points to its potential ability to illuminate the pathways through which personality is associated with accidents. The significant negative correlations of Conscientiousness (−0.62) and of Agreeableness (−0.26) with the FP general score are consistent across nearly all FP factors. These consistent relationships might clarify the reason for the repeated negative association of Conscientiousness and Agreeableness with accidents (Clarke and Robertson, 2005; Beus et al., 2015). In contrast, both Neuroticism and Extraversion have different patterns of correlations across the FP factors. Neuroticism was positively correlated to Lapses, Disorganization-Errors, Temporal-Errors, and Procedural-Violations, yet simultaneously negatively correlated with Risks. Extraversion negatively correlated with Lapses and Disorganization-Errors, but at the same time, positively correlated with Risks. The contradictory relationships with different categories of behavioral failures might underlie the inconclusive evidence on the associations between Neuroticism, Extraversion and accidents. The picture that emerges from the data suggests that the FP could provide a powerful vehicle to assist in understanding the complex relationships between personality and human failures.

Additionally, consistent with Beus et al. (2015), our data stresses that in order to better understand the magnitude of personality’s associations with behavioral failures, researchers must employ an expansive set of personality traits incorporating both broad and facet levels. In our study, the broad trait of Openness was associated only with the Risks factor, but once focusing on the lower facet of Ideas, significant associations with Procedural-Violations, Non-compliance and Disorganization-Errors emerged.

### Limitations and Further Research

The findings presented here suggest six distinct categories of behavioral failure. However, the FP is at its initial stage of development, and the validation of any scale is a cumulative, ongoing process. Therefore, the structure of factors that have been identified is preliminary. Despite this limitation, it is our sincere hope that our multifaceted scale may stimulate enlightening research, assist in achieving a deeper understanding of the important and relevant phenomenon of behavioral failures, and lead to more effective intervention strategies. We invite other researchers to help us improve the FP, and believe that subsequent research will expose additional categories of failures. We suggest the following productive avenues for further research:

#### Expand Beyond Self-Reporting

The current findings are based entirely on self-reported behaviors and tendencies. Due to the inherent unreliability of accidents as a dependent measure and their inability to tap the overall criterion space (Guion, 2011), there are calls in the safety literature to focus research on the behavioral antecedents of accidents, rather than on actual accidents (Lawton and Parker, 1998). Additionally, self-reported accidents and objective road safety data correlate fairly strongly (de Winter et al., 2015), suggesting that the self-reported data is valid. Yet, we believe that in order to further understand how human behaviors contribute to accidents, future research should also incorporate objective measures of actual accidents and mishaps. Such a research program is underway. It utilizes a diary method, and consists of several phases, including an initial session with the FP and a 10-day period, in which participants complete daily reports on their actual failures and mishaps. 85 students have already completed the study. Our preliminary results suggest a significant link between FP scores and actual daily involvement in exceptional events (such as being late to class, late submission of assignments, misplacing car keys and forgetting to do something important). These types of studies may help link specific categories of behavioral failures to specific types of accidents.

#### Improve the Internal Consistencies of the Subscales

The internal consistency of some of the subscales (and in particular DE) is lower than desired. These low consistencies might be due to the length of the scales. For example, the DE contains only two items. The formula for the Cronbach’s alpha is: α = *K*^∗^ mean *r*/(1 + (*K* − 1) ^∗^ mean *r*. Thus, two factors influence the magnitude of α: *K* (the number of items selected to constitute the scale) and mean *r*. A small number of scale items would violate tau-equivalence and give a lower reliability coefficient. Hence it is common to find quite low Cronbach values (e.g., 0.50) for scales with less than 5 items. Longer scales give higher alpha values (Hinton et al., 2014). Despite the lower reliabilities of specific subscales, we feel at this stage, that it is important to share our findings with other researchers and invite them to help us improve the scale. We believe that subsequent research will refine the scale and expose additional categories of failures. For example, it is unclear whether the items of DE should be treated as a subscale or will be divided into two subscales upon addition of items.

#### Obtain Additional Samples

The procedure of data collection in the current studies were not homogenous. In study 1 participants were recruited online, through social media, professional forums, and by email. In study 2, the sample included both undergraduate psychology students who participated in the study as part of their academic requirements, and volunteers who were recruited online. Study 3 tested job applicants in the process of pre-employment screening for a variety of positions in a large Israeli corporation, which were tested in person. Although the same factor structure consistently emerged over several studies with different populations, it is mandatory to validate and refine the FP among additional populations. Most of our respondents were educated Israeli adults. Previous research (e.g., Broadbent et al., 1982; Könen and Karbach, 2020) has indicated that behavioral failures (and in particular cognitive failures) do not appear to be very closely related to intelligence, cognitive ability or educational level. Nevertheless, it may still be the case that the FP is related to these constructs. Future studies might establish such a relationship.

#### Explore the Relationship Between the Failures Proneness Questionnaire Scores and Failures in the Workplace

The items of FP describe common failures in daily environments. In future studies it could be instructive to explore the relationship between the FP scores and failures in the workplace, for various professional domains.

#### Identify Mechanisms Beyond Behavioral Failures

Although the identification of different categories of behavioral failures is informative, it is also important to illuminate the sources of these failures. More empirical work is needed to systematically explore the cognitive and motivational mechanisms underlying these failures.

#### Examine the Stability of the Failures Proneness Questionnaire

The divergent relationships between personality facets and distinct categories of behavioral failures imply that the FP (like CFQ) measures trait-like characteristics of individuals. Yet, in order to confirm this, future studies should examine the stability of the structure over time (test–retest reliability).

#### Examine Personality Profiles

Several authors suggested that certain individual differences might interact to produce differential effects (e.g., Herzberg, 2009). They further introduced the idea of personality prototypes, which are based on trait configurations. Subsequently, studies confirmed that personality profiles have a powerful and reliable predictive capability for accident involvement (Witt et al., 2002; Wallace and Vodanovich, 2003; Sommer et al., 2008). Future studies could explore the relationships between distinct personality profiles and the FP. Additionally, the associations of personality and behavioral failures vary by age (Sutin et al., 2020). Hence, future research could examine whether the associations are moderated by age.

## Conclusion

The current research is a first attempt at developing and validating the Failures Proneness questionnaire − a multidimensional measuring instrument of an individual’s propensity toward behavioral failures, encompassing a wide variety of contexts. Our data confirms that the scale is reliable and yet covers diverse manifestations of behavioral failures in everyday environments. The significance of the FP lies in its potential ability to identify particular categories of behaviors that serve as distinct pathways to accidents. In view of the lack of a scale explicitly developed from a multidimensional perspective, we believe that our scale could offer new theoretical insights and yield important practical contribution. It could allow for greater variability in determining the specific focus for intervention when failures do occur. The theoretical significance FP stems from the FP’s potential to provide parameters upon which research outcomes can be compared and analyzed. Its practical importance lies in from its potential to provide important information for occupational screening and for the investigation of both accidents and exceptional events, as well as for guiding improvement. We hope that this research is the first of many, which will continue to generate valuable information for the future control of accidents.

## Data Availability Statement

The raw data supporting the conclusions of this article will be made available by the authors, without undue reservation.

## Ethics Statement

The studies involving human participants were reviewed and approved by Ethics committee of the Academic College of Tel Aviv-Yaffo. The patients/participants provided their written informed consent to participate in this study.

## Author Contributions

Both authors listed have made a substantial, direct, and intellectual contribution to the work, and approved it for publication.

## Conflict of Interest

The authors declare that the research was conducted in the absence of any commercial or financial relationships that could be construed as a potential conflict of interest.

## Publisher’s Note

All claims expressed in this article are solely those of the authors and do not necessarily represent those of their affiliated organizations, or those of the publisher, the editors and the reviewers. Any product that may be evaluated in this article, or claim that may be made by its manufacturer, is not guaranteed or endorsed by the publisher.

## Footnotes

1 Psiphas Psychological Application Ltd. was established in 1993. The company constructs and manages manpower evaluation systems. It develops and customizes tools (tests and questionnaires) to assess abilities, personality characteristics and integrity. The company has many customers, most of whom are large organizations. Over the years, hundreds of thousands were tested and evaluated by Psiphas’ products.
