# Supplementary material for: Measuring Failures Proneness: Scale Development and Preliminary Validations
Source: Front Psychol. 2021 Dec 13;12:757051. doi: 10.3389/fpsyg.2021.757051 (PMC8711769; doi:10.3389/fpsyg.2021.757051)
Supplement: Supplementary file 1 [file Presentation_1.pdf]

Appendix 1 - 30-item FP

The following statements describe everyday behaviors and situations. Please indicate the degree to which each statement reflects you on a scale of 1-7, where 1 represents the answer ”never true about me”, and 7 the answer ”always true about me”. Please mark the number that best describes you.

1 It happens that I text a message or fiddle with the mobile while driving.
2 When selling a car or any other item it is important for me to report all its defects.
3 I have moments of tiredness and lack of energy during the day.
4 It happens that I am unrealistic regarding arrival time estimation.
5 It happens that I forget my mobile at places where I have been.
6 After something distracts my attention, I can quickly return to what I was doing before.
7 It happens that after reading a paragraph I need to read it again because I was not concentrating.
8 I tend to be late or to arrive at the last moment.
9 It happens that I trust my luck.
10 I take risks hoping to advance myself in life.
11 I like doing things that lead to suspense and excitement.
12 I am punctilious and precise in what I do.
13 I work according to a diary and a detailed timetable which I prepare.
14 I adhere to all traffic rules while driving.
15 It happens that I send a WhatsApp message to the wrong person.
16 I try to collect all relevant data before deciding.
17 I tend to “round corners” in order to further my work and to finish on time.
18 When in conversation I remember what I intended to say even if I did not say it in real time.
19 It happens that I delay important matters to the last minute.
20 It happens that I do not adhere to a certain rule/instruction at work because it appears irrelevant.
21 I have difficulty in following what is said in a meeting with numerous participants.
22 It happens that I go somewhere without remembering what for when reaching it.
23 I daydream.
24 I tend to attach utmost importance to adherence to rules and directives.
25 It happens that I ignore accepted instructions in order to complete a task faster.
26 I respect authoritative figures and tend to accept authority easily.
27 I respect speed limits even at times when the streets are empty.
28 Routine is cumbersome for me which is why I need change and variety.
29 I familiarize myself carefully with the practices of a new place to which I am heading (foreign country, new workplace etc.).
30 I manage to keep my possessions (wallet, earphones, keys etc.) for a long time without losing them.
