# Supplementary material for: Measuring Failures Proneness: Scale Development and Preliminary Validations
Source: Front Psychol. 2021 Dec 13;12:757051. doi: 10.3389/fpsyg.2021.757051 (PMC8711769; doi:10.3389/fpsyg.2021.757051)
Supplement: Supplementary file 2 [file Presentation_2.pdf]

Appendix 2 - 16-item FP

The following statements describe everyday behaviors and situations. Please indicate the degree to which each statement reflects you on a scale of 1-7, where 1 represents the answer ”never true about me”, and 7 the answer ”always true about me”. Please mark the number that best describes you.

1. It happens that I am unrealistic regarding arrival time estimation.
2. It happens that after reading a paragraph I need to read it again because I was not concentrating.
3. I tend to be late or to arrive at the last moment.
4. I take risks hoping to advance myself in life.
5. I like doing things that lead to suspense and excitement.
6. I am punctilious and precise in what I do.
7. I work according to a diary and a detailed timetable which I prepare.
8. I adhere to all traffic rules while driving.
9. I tend to “round corners” in order to further my work and to finish on time.
10. It happens that I do not adhere to a certain rule/instruction at work because it appears irrelevant.
11. I have difficulty in following what is said in a meeting with numerous participants.
12. It happens that I go somewhere without remembering what for when reaching it.
13. I daydream.
14. I tend to attach utmost importance to adherence to rules and directives.
15. It happens that I ignore accepted instructions in order to complete a task faster.
16. I familiarize myself carefully with the practices of a new place to which I am heading (foreign country, new workplace etc.).
